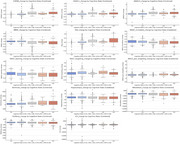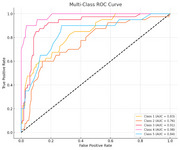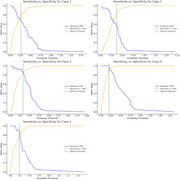# Longitudinal Multi‐Domain Biomarkers and Machine Learning for Predicting Alzheimer's Disease Progression

**DOI:** 10.1002/alz70858_101530

**Published:** 2025-12-25

**Authors:** Abraham Varghese, Vinu Sherimon, Ben George Ephrem

**Affiliations:** ^1^ University of Technology and Applied Sciences, Alkhuwair, Muscat, Oman; ^2^ University of Technology and Applied Sciences, Muscat, Muscat, Oman; ^3^ University of Technology and Applied Sciences, Muscat, Oman

## Abstract

**Background:**

Alzheimer's disease (AD) is characterized by progressive cognitive decline, memory impairment, and neurodegeneration. This study examines both stable and transition stages of AD progression, utilizing baseline and follow‐up data to derive rate‐of‐change measures. By integrating clinical, memory, and imaging biomarkers, a multi‐domain approach was adopted to predict transitions and assess disease progression. Sensitivity analysis highlights the model's capability to detect transitions effectively, while specificity minimizes false alarms, ensuring accurate identification of at‐risk individuals. The goal is to determine whether a patient's condition will progress to the next stage or remain stable, enabling timely and personalized therapeutic interventions.

**Method:**

This longitudinal study analyzed data from 1,416 patients categorized into stable (CN, MCI, AD) and transition groups (CN‐to‐MCI, MCI‐to‐AD). Missing data (<5%) were imputed, and patients with >10% missing data were excluded. Continuous variables were normalized, and clinically plausible outliers were retained. Key variables were grouped into cognitive, memory, and imaging domains and assessed using pairwise t‐tests. Logistic Regression was unsuitable due to multicollinearity and non‐normal residuals. A Random Forest Classifier, selected for its ability to model non‐linear relationships, was trained (80%) and tested (20%) on stratified data. The model was evaluated using Accuracy, ROC‐AUC, and sensitivity‐specificity analyses to assess predictive performance.

**Result:**

Figure 1 reveals significant trends across cognitive, memory, and imaging biomarkers. Cognitive measures (e.g., CDRSB_change, ADAS11_change) increased with severity, while memory measures (e.g., RAVLT_immediate_change) declined, with MCI‐to‐AD transitions showing the steepest changes. Imaging biomarkers, such as Ventricles_change and Hippocampus_change, indicated marked structural atrophy in transitions to AD. The Random Forest Classifier achieved strong performance, with AUC values of 0.76–0.98 (Figure 2), excelling in MCI‐to‐AD transitions. Sensitivity‐specificity analyses (Figure 3) highlighted a trade‐off, showcasing the model's ability to distinguish stable stages from transitions effectively

**Conclusion:**

This study demonstrates the effectiveness of machine learning with multi‐domain biomarkers in predicting Alzheimer's disease progression. The Random Forest Classifier achieved high AUC values, with key biomarkers like CDRSB_change and Hippocampus_change identifying transitions. This approach supports early interventions and personalized care, with future work focusing on longer follow‐ups and additional biomarkers.